# Engaging in a tone-detection task differentially modulates neural activity in the auditory cortex, amygdala, and striatum

**DOI:** 10.1038/s41598-017-00819-z

**Published:** 2017-04-06

**Authors:** Renjia Zhong, Lanlan Ma, Ling Qin

**Affiliations:** 1 Department of Emergency, First Affiliated Hospital, China Medical University, Shenyang, 110001 People’s Republic of China; 2 Department of Physiology, College of Basic Medical Science, China Medical University, No. 77 Puhe Road, Shenyang North New Area, Shenyang, Liaoning Province P.R. China 110122

## Abstract

The relationship between attention and sensory coding is an area of active investigation. Previous studies have revealed that an animal’s behavioral state can play a crucial role in shaping the characteristics of neural responses in the auditory cortex (AC). However, behavioral modulation of auditory response in brain areas outside the AC is not well studied. In this study, we used the same experimental paradigm to examine the effects of attention on neural activity in multiple brain regions including the primary auditory cortex (A1), posterior auditory field (PAF), amygdala (AMY), and striatum (STR). Single-unit spike activity was recorded while cats were actively performing a tone-detection task or passively listening to the same tones. We found that tone-evoked neural responses in A1 were not significantly affected by task-engagement; however, those in PAF and AMY were enhanced, and those in STR were suppressed. The enhanced effect was associated with an improvement of accuracy of tone detection, which was estimated from the spike activity. Additionally, the firing rates of A1 and PAF neurons decreased upon motor response (licking) during the detection task. Our results suggest that attention may have different effects on auditory responsive brain areas depending on their physiological functions.

## Introduction

Attentional engagement is an important determinant of how effectively we gather sensory information. Several human functional magnetic resonance imaging (fMRI) studies have shown that the activation of auditory cortex (AC) is modulated by listening tasks^1–3^ and attentional modulation is stronger outside the core regions of AC^4^. Many electrophysiological experiments have examined the effect of attention on neural activity in the AC of animals, albeit the observations remain controversial. Indeed, most studies have reported increased sound-driven neural responses when animals are actively engaged in an auditory behavioral task, compared to passively listening to auditory stimuli^5–7^. In contrast, other studies have reported either a suppression of the neural response due to task-engagement^8^ or no change between the engaged and passive conditions^9, 10^. Previously, we have systemically examined the effects of task-engagement on neural activity in different regions of the cat’s AC and found both increased and decreased spike activity in neurons of the primary AC (A1) during the active hearing period. However, the neural responses in the non-primary AC consistently showed increased spike activity that resulted in enhanced neural discrimination of sound stimuli^11^. Atiani *et al*.^12^ have also reported larger behavioral modulation of neural coding in the belt area of the AC compared with A1. These results suggest that attention could have different effects in different parts of AC. However, most electrophysiological studies on task-related changes have been conducted on the level of AC, while the subcortical structures have been less investigated.

Auditory sensitive neurons have been reported in subcortical structures like the amygdala (AMY) and overlaying areas of the striatum (STR)^13–17^. Anatomical studies have revealed a network of connections linking AMY and STR with the medial geniculate complex and the AC^18^. In addition, AMY shows increased responsiveness to a sound after association of that sound with an adverse stimulus (foot shock)^19–22^. Thus, these connections may be involved in processing auditory information with emotional/motivational significance^23–25^. Recent studies have demonstrated that projections from AC to STR participate in the transformation of an auditory sensation into motor response^26, 27^. The auditory responses in the AMY and STR have been well studied, but the attentional modulation of auditory response in those regions remains unknown. In the present study, we investigated the effect of attention on neural activity in AC, AMY, and STR of cats using a tone-detection task. We examined the difference between neural activity when the cats were attentively and passively listening to a set of pure-tone stimuli.

## Results

**Figure 1:**
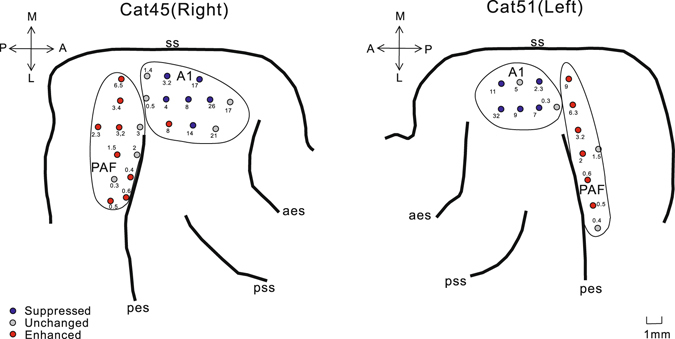
Reconstruction of the locations of recorded units in the AC of two representative cats. Different symbols mark the location of different groups of units. A1: primary auditory cortex; aes: anterior ectosylvian sulcus; PAF: post auditory field; pes: post ectosylvian sulcus; pss: pseudosylvian sulcus; ss: surasylvian.

**Figure 2:**
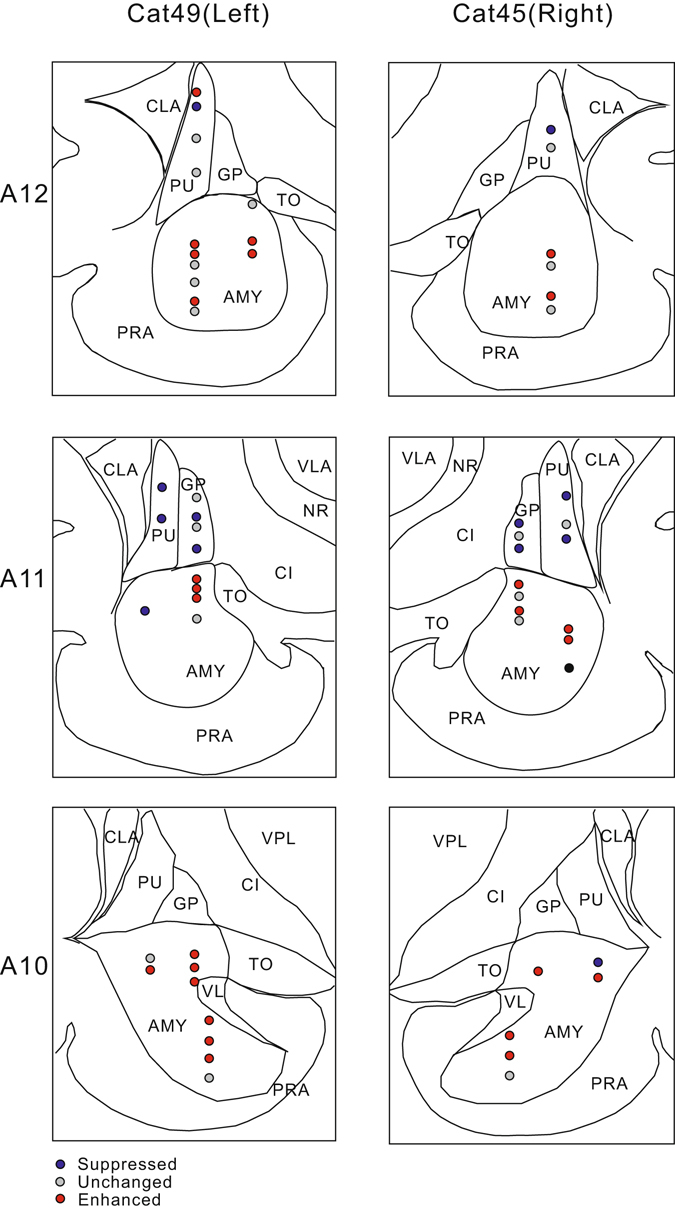
Reconstruction of the locations of recorded units in the AMY and STR of two representative cats. A10, A11, and A12 were 10, 11, and 12 mm anterior to the interaural plane, respectively. Different symbols mark the location of different groups of units. AMY: amygdala; CI: capsula interna; CLA: claustrum; GP: globus pallidus; NR: nucleus of reticularis thalamus; PRA: cortex of pariamygdalae; PU: putamen; STR: striatum; TO: tractus opticus; VL: lateral ventriculus; VLA: lateral ventral thalamus; VPL: posterior lateral ventral thalamus.

We recorded single-unit spike activity of 293 units from the AC, AMY, and STR in both hemispheres of four cats while they were actively engaged in the tone-detection and passive listening to the tone stimuli tasks. Figures 1 and 2 illustrate the locations of recorded units in the AC, AMY, and STR of two representative cats. In AC, our samples focused on the A1 and posterior auditory field (PAF). Given that there was no significant difference between the data collected from different cats, we pooled the data across animals and compared the neural responses to pure-tone stimuli under different conditions. We found that task-engagement had specific and varied effects on the neural response as presented below.

### Effects of task-engagement on pure-tone evoked responses

**Figure 3:**
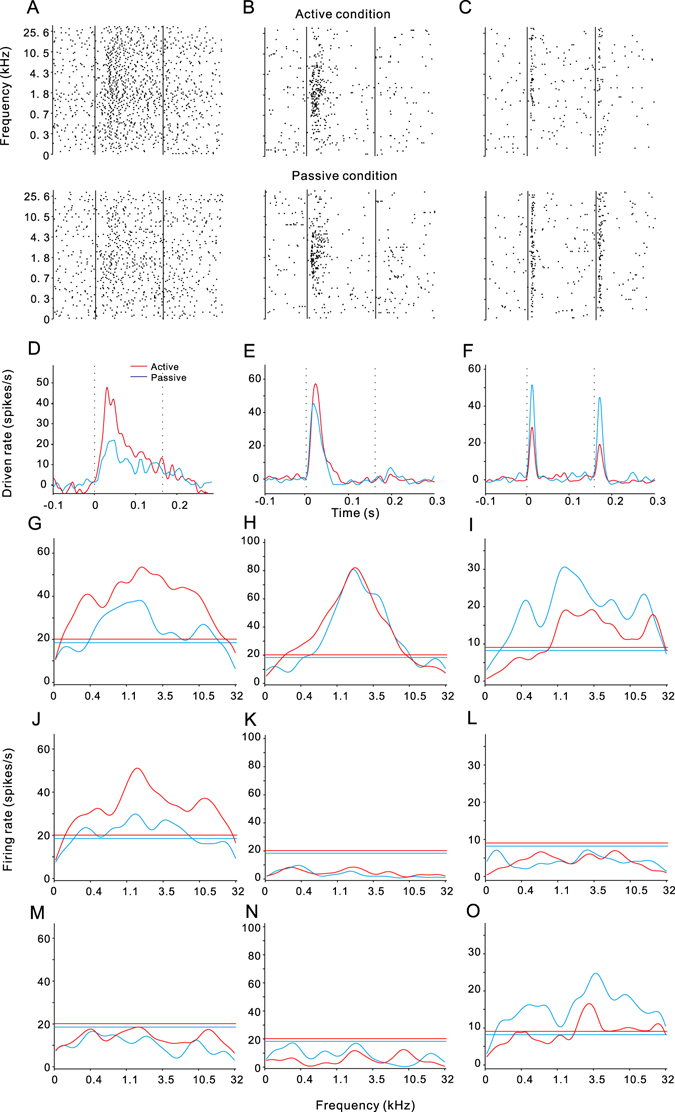
Examples of tone-evoked neural responses affected by task-engagement. (**A**–**C**) Raster displays of spike discharges in response to pure-tone stimuli at 125 different frequencies. Top and bottom panels show the results under the active and passive conditions, respectively. The two vertical lines mark the onset and offset of sound stimuli. Data in (**A**) were recorded from PAF, (**B**) from A1, (**C**) from PU. (**D**–**F**) Peri-stimulus time histograms (PSTHs) constructed from the raster displays under different conditions. The two vertical lines mark the onset and offset of sound stimuli. (**G**–**I**) Frequency tuning curves of onset segment, in which firing rate counted from 0 to 50 ms after stimulus onset is plotted against tone frequency. Horizontal lines show the thresholds to calculate BW. (**J**–**L**) Frequency tuning curves of ongoing segment (50 to 160 ms after stimulus onset). (**M**–**O**) Frequency tuning curves of offset segment (0 to 50 ms after stimulus offset).

Figure 3 shows representative responses of three single units driven by pure-tone stimuli under the active and passive conditions. The plots in Fig. 3A–C present a raster display of spike times in response to 128–32,000 Hz pure-tones at 60 dB sound pressure level (SPL). The tone-evoked responses in the neuron shown in Fig. 3A (recorded from the PAF) were clearly enhanced under the active condition (upper panel) compared with those under the passive condition (lower panel). To quantitatively compare the temporal pattern of neural response under different conditions, we plotted the peri-stimulus time histogram (PSTH) by counting the driven rate of spike activity across all tested pure-tone frequencies (Fig. 3D). In the passive session, the PSTH of this unit exceeded the threshold (2 standard deviations [SD] of background firing rate) at 15.8 ms after the tone onset and reached the peak height of 22 spikes/s. In the active session, it increased to 48 spikes/s and the response latency shortened to 10.6 ms (Fig. 3D). We calculated the modulation index (MI) to quantify the effects of task-engagement on the peak magnitude of PSTH (see Methods). The MI of this unit was 0.37. Because it was higher than the 95% confidential interval (CI), estimated by a bootstrapping method (see Methods), we deemed that the response magnitude of this unit was significantly enhanced by the task-engagement.

The spectral response of the recorded unit was analyzed in three time windows: “onset” (0–50 ms after stimulus onset), “ongoing” (50 ms from stimulus onset to stimulus offset), and “offset” response (0–50 ms after stimulus offset). The tuning curve of each time window was constructed by plotting the average firing rate in the “onset”, “ongoing”, or “offset” segment, respectively, against tone frequency (Fig. 3G). The bandwidth (BW) of frequency tuning in each segment was defined as the frequency range where the tuning curve was higher than the mean + 2 SD of the background firing rate (horizontal line). The BW_onset_ was extended from 5.8 to 7.2 octaves by task-engagement, while BW_ongoing_ was extended from 5.2 to 7.3 octaves. This unit did not show a BW_offset_ in both the active and passive session. Best frequency (BF) was estimated as the frequency at which the tuning curve reached its maximum. The BF of this unit remained relatively stable (1.8 kHz) across the experimental sessions. Taken together, the tone-evoked responses of this unit were enhanced by task-engagement in both temporal and spectral domains.

We also observed a case where the spike activity was changed to a lesser extent by task-engagement (Fig. 3B, recorded from the A1). The PSTHs and tuning curves during different sessions were similar in this unit (Fig. 3E,H,K and N). The tone-evoked responses were also suppressed by task-engagement in a unit recorded from the putamen (PU) (Fig. 3C). The peak magnitude of PSTH in this case decreased from 52 to 29 spikes/s (MI = −0.28, <95% CI), whereas the response latency was extended from 5.2 to 11.3 ms (Fig. 3F). The BW_onset_ decreased from 7.3 to 5.1 octaves and BW_offset_ decreased from 7.6 to 3.9 octaves (Fig. 3I,L and O).

### Effects of task-engagement on the population responses to a pure tone

**Figure 4:**
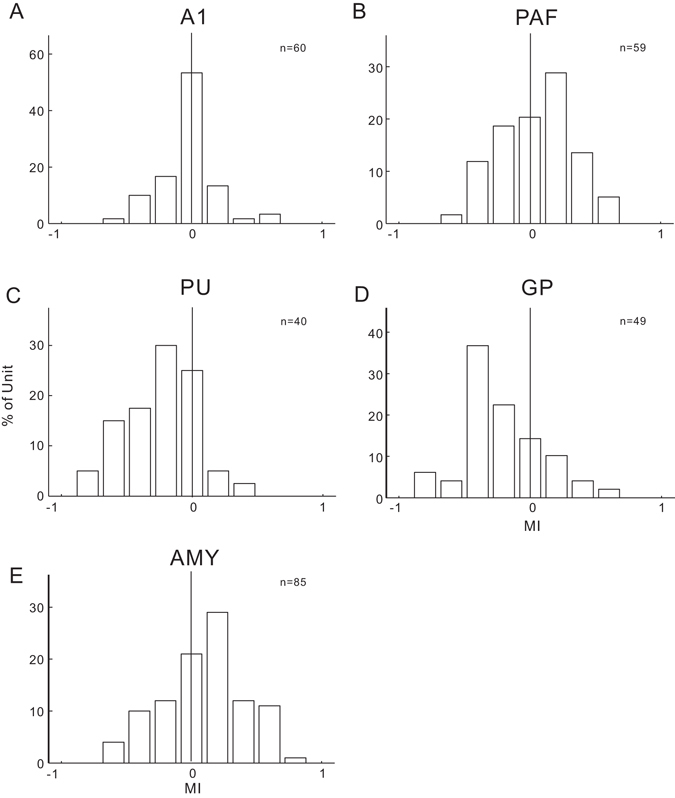
Distribution of the modulation index (MI) among the recorded units in different brain areas.

**Figure 5:**
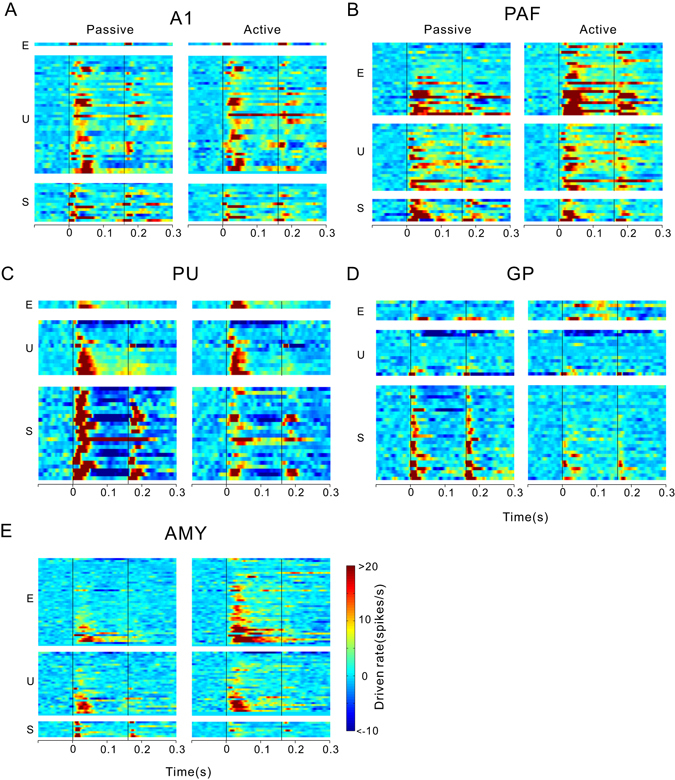
Effects of task-engagement on the tone-evoked responses in the neural population of different brain areas. Individual peri-stimulus time histograms (PSTHs) of enhanced (E), unchanged (U), and suppressed (S) units are aligned at stimulus onset and displayed in color-scaled plots. Black vertical lines show stimulus onset and offset.

We analyzed the MI distribution in neurons recorded in different brain areas (Fig. 4). The units were divided into enhanced, unchanged, and suppressed groups based on whether the MI deviated significantly from zero (> or <95% CI, see Methods). Table 1 shows the number (percentage) of different groups of units in different brain areas. The individual PSTHs of the enhanced, unchanged, and suppressed units are illustrated in the color-scaled plots in Fig. 5.

**Table 1:** Number (percentage) of different types of units in different brain areas.

	AMY	PU	GP	A1	PAF
Enhanced	44 (51.8%)	2 (5.0%)	6 (12.2%)	1 (1.7%)	26 (44.1%)
Unchanged	32 (37.7%)	14 (35.0%)	14 (28.6%)	44 (73.3%)	24 (40.7%)
Suppressed	9 (10.6%)	24 (60.0%)	29 (59.2%)	15 (25.0%)	9 (15.3%)

The MI in A1 was close to zero with a median of −0.003 (Fig. 4A), indicating that the tone-evoked responses in most of the A1 units were not changed by engaging in the behavioral task. Nevertheless, the number of units showing negative MI (suppressed unit) was higher than that showing positive MI (enhanced unit, Table 1 and Fig. 5A). The MI was distributed more widely in PAF than in A1 (Fig. 4B). The number of enhanced units was bigger than the suppressed units (Table 1 and Fig. 5B). The median of MI distribution in PAF was 0.09, suggesting that task-engagement had an overall enhanced effect on PAF neurons. The MI distribution in the PU and globus pallidus (GP) was biased toward negative with median value of −0.20 and −0.28, respectively (Table 1 and Fig. 4C,D). More than the half of the neural responses in PU and GP were suppressed under the active condition compared with the passive condition (Table 1 and Fig. 5C,D). In contrast, the median of MI was positive (0.12, Fig. 4E) in AMY, where around half of the neural responses were enhanced under the active condition (Table 1 and Fig. 5E). Figures 1 and 2 illustrate the spatial organization of the different types of units shown in two example cats.

### Effect of task-engagement on the properties of neural response

**Figure 6:**
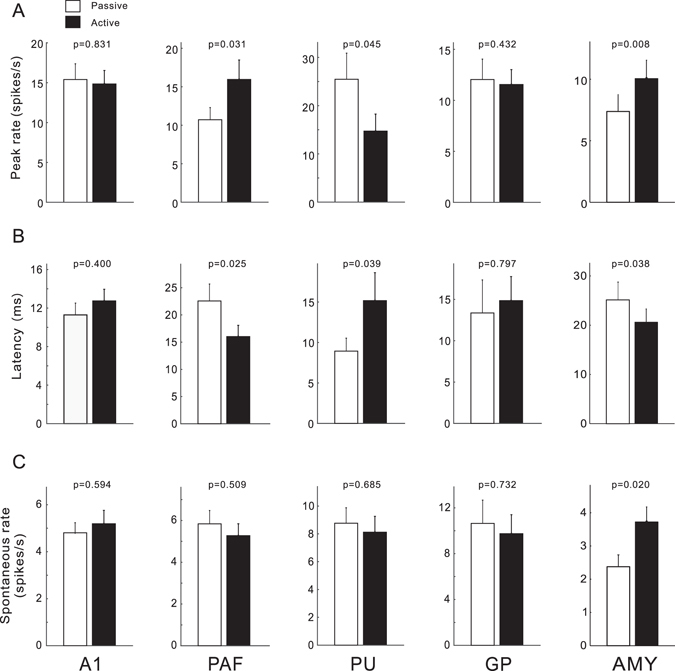
Comparison of the peak firing rate, response latency, and spontaneous rate under different conditions. Histograms represent means, and bars represent standard errors. Numbers are the *p* values of the paired *t*-test between mean values of the active and passive sessions.

Subsequently, we measured the peak rate, response latency, spontaneous rate, and BW in the units recorded from A1 (n = 60), PAF (n = 40), PU (n = 40), GP (n = 49), and AMY (n = 85) of four cats, and examined whether task-engagement significantly changes the properties of neural response in each brain area. For this, we conducted a paired *t*-test between the passive and active sessions. As shown by the mean and standard error (SE) in Fig. 6A, the peak rate of PAF and AMY units during the active session was significantly increased comparing to the passive session (*p* < 0.05), while the peak rate of PU units was significantly decreased. No significant change was observed in the peak rates of A1 and GP units. Task-engagement significantly extended the neural response latencies in PU, but shortened them in PAF and AMY (Fig. 6B). Moreover, the spontaneous rate in AMY increased by task-engagement. However, there was no significant effect on the other brain areas (Fig. 6C).

**Figure 7:**
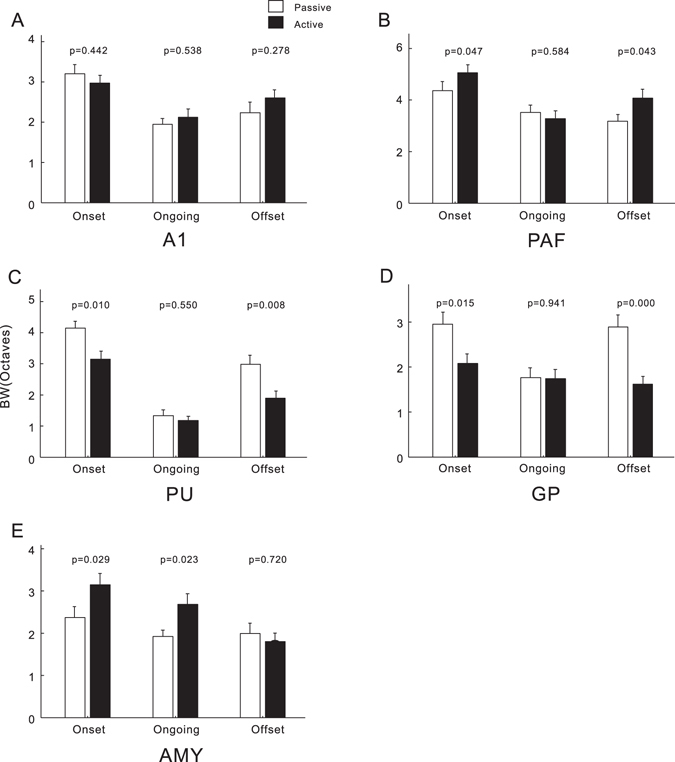
Comparison of the bandwidths (BW) of the tone-evoked response under different conditions. Conventions are as described in Fig. 6.

Figure 7 shows the comparisons of BW in the units of each brain area. The BWs were not significantly affected by task-engagement in A1 (Fig. 7A). In PAF, BW_onset_ and BW_offset_ significantly increased (*p* < 0.05, paired *t*-test, Fig. 7B), while they significantly decreased in PU and GP (Fig. 7C,D). In AMY, task-engagement significantly increased BW_onset_ and BW_ongoing_ (Fig. 7E). In contrast, BF was not significantly altered by task-engagement in all the examined brain areas (data not shown).

In summary, the enhanced effects of task-engagement included an increase in firing rate, reduction of response latency, and extension of BW. However, the suppressed effects included a decrease in firing rate, delay of response latency, and shrinkage of BW. The enhanced effect was commonly observed in PAF and AMY, while the suppressed effect was dominant in PU and GP.

### Effect of task-engagement on the neural detection of sound stimulus

**Figure 8:**
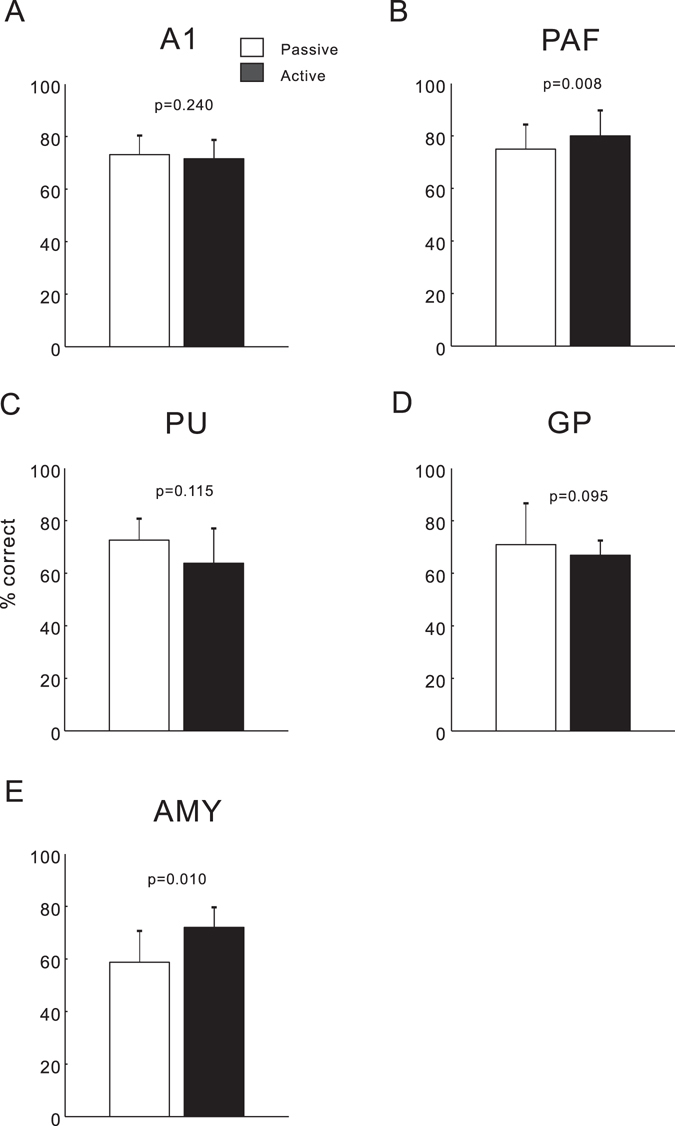
Comparison of accuracy of tone detection based on the neural spikes of active and passive sessions. Conventions are as described in Fig. 6.

We further conducted a neurometric analysis to estimate the rate of sound detection, when an ideal observer detected the tone stimuli based on the neural spikes (see Methods). Figure 8 shows the mean and SE of the neurometrics of each brain areas during passive and active sessions. We observed a significant improvement in the correct percentage of neural detection of sound stimulus in the PAF and AMY units during active sessions (*p* < 0.01, paired *t*-test), indicating an increased detection signal encoded by spike activity. In the PU and GP units, accuracy of neural detection decreased during active sessions; however, the change was not significant.

### Effect of licking response on the neural activity

**Figure 9:**
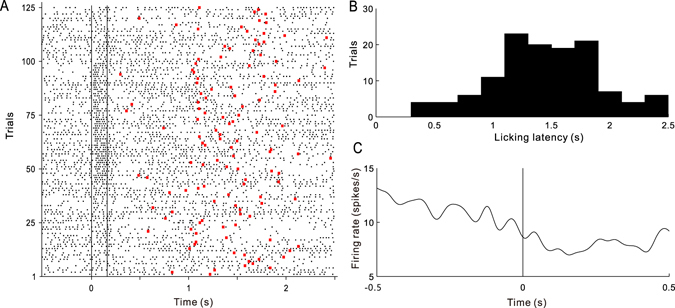
Effect of licking response on the spike activity of the auditory neuron. (**A**) Raster plot of an example unit in an active session. Red dot represents the the time of first licking. (**B**) Distribution of the latency of the first licking. (**C**) Peri-licking peri-stimulus time histogram (PSTH). Vertical line represents the time of the first licking.

**Figure 10:**
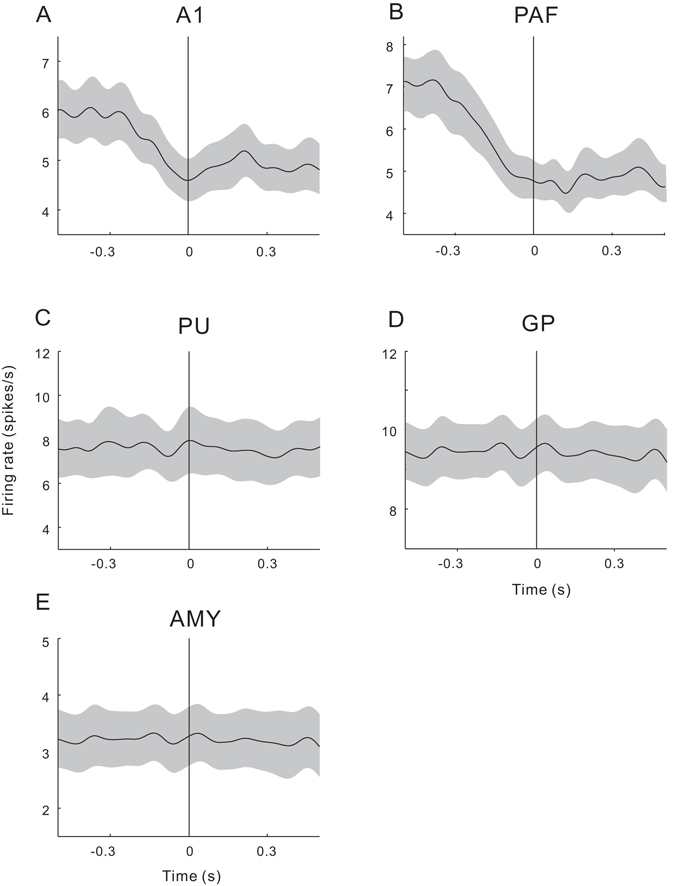
Peri-licking peri-stimulus time histograms (PSTHs) averaged across the units in different brain areas. Shaded area represents the standard error. Vertical line represents the time of the first licking.

Finally, we examined how the cat’s licking response affected the spike activity of auditory neurons. Figure 9A shows the raster plot of a unit recorded during the active session. The time point when the cat started to lick in each trial was marked by a red dot on the raster plot. As it has been trained, the cat mostly started to lick at 1–2 s after the tone stimulus (Fig. 9B). We selected the trials in which the licking latency was between 1–2 s to avoid an overlap between tone-evoked response and licking movement. We aligned the spikes to the start time of licking response, and then constructed the peri-licking PSTH (Fig. 9C). The firing rate showed a gradual decline before the start of licking, and remained low during the licking response. The mean and SE of peri-licking PSTH of each brain area is presented in Fig. 10. We found that the firing rates of A1 and PAF decreased before the licking response (Fig. 10A,B), while those of PU, GP, and AMY were not modulated by the licking response (Fig. 10C,D).

## Discussion

In the present study, we examined the behavioral effects of tone-detection task on the AC. Recent pharmacological inactivation and optogenetic silencing studies showed that AC can modulate the sensitivity of sound detection in behavioral tasks^28, 29^. We found that the basic properties of neural responses (peak firing rate, response latency, spontaneous rate, and BW) were less affected by task-engagement in A1. A previous study has reported that spatial tuning of A1 neurons sharpened when a cat engaged in a location-relevant task^30^. The difference in the change of tuning may originate from the difference of task demand: the detection task used in our study and discrimination task in the previous study. It has been noted that the modulating effects of behavioral task can vary according to the task’s difficulty or type. The enhanced neural responses during the tone-detection task could change to suppressed responses during other tasks and vice versa^11, 31^. Furthermore, the behavioral meaning of a stimulus (shock vs. reward) can drive neural responses in opposite directions^32^. Thus, the characteristics and demands of tasks in different studies may cause different effects of task-engagement.

On the contrary, several studies have used the same behavior to investigate neural responses in multiple areas of the AC and suggest that task-engagement tends to enhance the activation of higher stages in AC^11, 12^. Consistent with this, our results show that task-engagement enhancement was more commonly observed in PAF than A1. Indeed, PAF is located at a higher stage of the auditory hierarchy than A1, as it has a longer response latency and more complex response patterns than A1^33–35^. This view is further supported by the fact that cooling of A1 is followed by a decrease in response and sharpened spectral tuning in PAF, whereas the activity in A1 is not altered by cooling of the PAF^36^.

Both AMY and STR are not traditionally viewed as auditory processing structures; however, some studies have reported many of the neurons in these subcortical areas to be responsive to auditory stimuli^13–17^. Indeed, AMY and STR play significant roles in a multitude of functions, including reward processing, emotional learning, and goal-directed response^23–25, 37^. Thus, it is not surprising that their activity is sensitive to a change in behavior. Our study demonstrates that engaging in a tone-detection task generates different effects in AMY and STR. Tone-evoked responses were enhanced in AMY and suppressed in STR. The enhanced effects were indicated by an increase of the firing rate, shortening of response latency, and extension of BW of the tuning curve. In contrast, the suppressed effects included a decrease in firing rate, delay of response latency, and shrinkage of BW. Such an enhanced auditory response can increase the accuracy of tone-detection when an ideal observer makes a decision based on the neural activity.

The contrasting effects of attentive listening on AMY and STR may relate to the differences in their physiological functions. It is well established that AMY mediates fear-related attention towards signals such as threat under a stressful circumstance^38^ and under auditory fear conditioning^23, 39^. As the responses of AMY neurons to a sound markedly increase after the sound becomes conditioned to fear^19–22^, the auditory responsive neurons may be involved in establishing associations between auditory cues and biologically important events, and orchestrating emotional responses. The STR areas (PU and GP) examined in this study are basal ganglia involved in motor, oculomotor, executive, and limbic functions^40, 41^. Previous studies have suggested that basal ganglia mediates the acquisition and performance of goal-directed instrumental actions^25^. It has also been shown that many striatal neurons are responsive to sound stimuli^13, 14, 17^, and that stimulus selectivity of striatal neurons is similar to AC neurons^26^. Auditory representations in the STR may be integrated with information from other modalities and relayed to higher motor centers to elicit appropriate actions^42–44^. Future research is necessary to determine the roles of auditory attention in different physiological functions of AMY and STR.

We found that the firing rate of AC neurons decreased before and during the licking response. Because the cats were required to lick at 1–2 s after the sound presentation in our task, the licking-related effects do not explain the difference of tone-evoked neural responses between the active and passive states. Our results indicate a modulation of movements on the spontaneous firing rate of auditory neurons. This is consistent with a previous study of freely behaving mice reporting that the membrane potential dynamics of excitatory AC neurons are suppressed immediately before and during a variety of movements including locomotion, head movements, and other body movements such as grooming^45^. It has also been demonstrated that sound-evoked responses in the AC are suppressed during walking^46^ or vocalization^47, 48^. The motor-related modulation may be mediated by a direct projection from the motor cortex to the AC, which is a common feature of the mammalian brain^49, 50^. This is supported by the evidence that a heightened motor cortical activity correlates with auditory cortical suppression in humans^51^, and directly activating motor cortical synapses suppresses spontaneous and stimulus-evoked responses in anaesthetized mice^52^. A recent study using electrophysiological and optogenetic methods reveal that motor-related changes in AC are driven by a subset of neurons in the secondary motor cortex^45^. The physiological significance of the motor-related suppression has been proposed to transiently dampen cortical sensitivity to predictable sounds, enabling AC neurons to maintain response to unexpected stimuli^46^.

On the other side, our results indicate that licking was not associated with significant effects in AMY and STR. This was unexpected, as AMY and STR have been reported to associate with goal-directed action and reward expectation^25, 53–55^. This observation may be attributed to the specific conditions of our study. First, we only selected the units responsive to tone stimuli, which may be less associated with movement. Second, the cats had been over-trained by the tone-detection task before being subjected to the electrophysiological recording. It was found that the motor-related response in the STR decreased with increased training^56^. Third, because the primary objective of this study was to investigate the change of tone-evoked response under different behavioral states (passive and active listening), we did not design the trials with unexpected more or less reward in the behavioral paradigm, which would have been more effective to activate AMY and STR^53–55^. Thus, a complex behavioral task is needed to investigate the neural mechanisms about motor and reward.

In conclusion, we found that engaging in a tone-detection task has a promoting effect on the neural activities in PAF and AMY, which may be associated with an improvement in tone detection. In contrast, the tone-evoked neural responses are suppressed in PU and GP. Moreover, tone-evoked neural activity in A1 and PAF decreased upon motor response. Thus, our results indicate that the present attention-engaging task can differentially modulate different regions of the brain.

## Methods

### Ethics statement

All experimental protocols were approved by the China Medical University Animal Care and Use Committee and were in strict accordance with the National Institutes of Health Guide for the Care and Use of Laboratory Animals (NIH Publications No. 80–23), revised in 1996. All surgeries were performed under sodium pentobarbital anesthesia, and all efforts were made to minimize animal suffering.

### Apparatus

The behavioral training and electrophysiological recording experiments were conducted in an electrically shielded, sound-attenuated chamber. Custom-built software in MATLAB (Mathworks Inc., Natick, MA, USA) interacted with the apparatus via digital input-output hardware (PCI-6052E; National Instruments, Austin, TX, USA). Auditory stimuli were digitally generated by custom-built software and delivered via a pair of speakers (K701; AKG Acoustics, Vienna, Austria) placed 2 cm from the cat’s auricles. Sound was calibrated using a Bruel & Kjaer 1/2″ condenser microphone with a 2669 preamplifier positioned at the cat’s ear meatus. The system frequency transfer function was flat up to 32 kHz (±6 dB). A video camera and a photoelectric sensor were used to monitor the cat’s behavior.

### Training paradigm and behavioral tasks

Adult female cats (n = 4) were trained for a pure-tone-detection task. During the training, cats were placed in a customized-design training frame and wrapped in a cotton bag to keep them from scratching the devices with their claws. A metal pipe was set in front of the cat’s mouth to deliver liquid food. The cats were deprived of food to 80–90% of their free-feeding body weight, but had free access to water. The cats were habituated to the experimental setting and trained to obtain food by licking the metal pipe. Subsequently, cats were trained to lick the pipe within a limited time window after a tone burst was presented. The cats would obtain a drop of food from the pipe as a reward if they licked within 1–2 s after a tone was presented (correct response). Licking too early or too late was recorded as an incorrect response and caused a time-out. A new tone presentation trial was not triggered until the cats had consumed the reward and stopped licking for several seconds (4–8 s, depending on the cat’s performance in previous trials). Such a behavioral setting ensured that the cats attended to the tone presentation. The tone bursts were 60 dB at sound pressure level and 160 ms in duration with a 5 ms rise/fall time. One session consisted of 125 tone bursts at different frequencies of 0.1–32 kHz in logarithmic scale. The cats demonstrated a reliable response to detect tones after 40–50 sessions by reaching a correct response rate of 80% in three consecutive sessions. Thereafter, cats underwent surgery for the electrophysiological recording experiments.

### Surgery

A stainless steel head-post was surgically implanted in the skull for stable electrophysiological recording. During surgery, the cats were anesthetized with sodium pentobarbital (30 mg/kg) and fixed to a stereotaxic frame (SN-3N; Narishige International Inc., East Meadow, NY, USA). Using a sterile procedure, the skull was surgically exposed, and the head-post was mounted on the occipital bone using stainless steel screws. A plastic chamber (inner diameter, 25 mm) was mounted on the skull for microelectrode access to the AC or AMY and STR. The head-post and recording chamber were implanted with bone cement. Antibiotics and analgesics were administered as needed following surgery. After recovery from the implantation surgery (2–3 weeks), the cats were habituated to head restraint in a customized holder over 1–2 weeks and then trained to the task criterion for additional 2 weeks while restrained in the holder.

### Neurophysiological recording

Experiments were conducted in a double-walled, sound-attenuated chamber. A 0.5 mm diameter hole was drilled into the skull, the dura was pierced with a sharp probe, and a single epoxylite-insulated tungsten microelectrode (FHC Inc., Bowdoin, ME, USA; impedance: 2–5 MΩ at 1 kHz) was advanced into the brain using a remote-controlled micromanipulator (MO-951; Narishige). The approach angle of the electrode during penetration was perpendicular to the brain surface for AC recording or along the vertical axis for STR and AMY recording. The coordinate of each electrode site was calibrated to a fixed mark inside the recording chamber. Electrode output was amplified using a high impedance head-stage and a preamplifier (RA16AC and RA16PA; TDT Inc., Alachua, FL, USA). The output of the preamplifier was delivered to a digital signal processing module (RX-7; TDT), and band-pass filtered at 500–3,000 Hz. The neural waveforms were digitized and stored on a computer hard disk using OpenEx software (TDT) for analysis.

After the microelectrode penetrated to the target brain area and tone responsive spikes were stably isolated, we started the behavioral procedure to record a session of spike activity as the cats listened actively to a 125 pure-tone stimuli. Subsequently, we withdrew the food-delivery pipe and recorded the spike activity as the cats passively listened to the same stimuli. The inter-stimulus interval during the passive session was the same as that during the active session. After collecting sufficient data from one recording site, the electrode was moved forward to search for another unit. We collected 1–3 units during 1 day of experiments lasting for 3–5 h.

### Data analysis

The spikes were sorted offline using OpenSorter software to include only single-unit spikes in the analysis. Spike activity driven by pure-tone stimuli was aligned along the stimulus onset to construct a raster plot of each tone frequency (Fig. 3A–C). A PSTH, which counts the spikes across the 125 trials of different frequencies, was computed with a 1-ms bin width and smoothed with a Gaussian function and 5 ms standard deviation (Fig. 3D–F). The background corresponded to the spontaneous firing rate detected 500 ms before stimulus onset. The PSTH height was transformed into the “driven rate” by subtracting the background firing rate. Response latency for each unit was defined as the time at which the PSTH was higher than 2 SD of the background firing rate. Response magnitude was assessed based on the maximum driven rate of the PSTH.

To quantify the effects of task-engagement on the tone-evoked neural responses, we calculated modulation index (MI) for each unit using the following equation:

*MI* = (*A* − *B*)/(*A* + *B*), where A and B are the peak height of the PSTH during active and passive sessions. Since the peak heights of PSTH in both sessions were positive, MI varied between +1 and −1, where positive and negative signs indicate the enhancement and suppression of neural response by task-engagement, respectively.

To determine if task-engagement significantly modulated the neural response, we used the bootstrapping method to test if the MI of each neuron significantly differed from zero. The recording trials for the active and passive sessions were shuffled and divided randomly into two groups (A and B) to calculate the MI. This procedure was repeated 1,000 times to estimate the 95% CI of the MI. Units with positive MI > 95% CI were deemed as “enhanced”, whereas those with a negative MI < 95% CI were deemed as “suppressed” units. The remaining units were “unchanged”.

Neurometric analysis based on the spike distance metric was used to test responses of a neuron for detection of tone stimuli^57–59^. For each neuron, a PSTH during the 0–210 ms post-stimulus period of one trial was chosen and removed from the data, referred to as the test trial. A template PSTH was then constructed using the mean of the remaining trials. The segment from 0 to 210 ms and from −210 to 0 ms, after stimulus onset was considered as the template of sound-driven and background PSTH. Subsequently, we compared how similar the test PSTH was to the sound-driven and background PSTH templates. For this, we calculated the Euclidian distance (ED) between the test and template PSTH, which is the square root of the sum of squared differences between firing rates at each bin (i).

$${\rm{ED}}=\sqrt{\sum _{i=1}^{{n}_{bin}}{({x}_{i}-{y}_{i})}^{2}}$$

where, n_bin_ is the total number of bins and x, y are bin heights.

If the test and template PSTH were similar, then ED would be small, whereas if they were different, then ED would be large. The test PSTH was assigned to be the sound-driven or background group that had the most similar PSTH template. This procedure was repeated until each trial of a neuron was considered as test data. The percentage of correct classification (% correct) was calculated as the percentage of the total number of trials in which the correct stimulus was selected. The chance level for classification was 50%, because a PSTH could be equally assigned to the sound-driven or background set when there was no difference between them.

### Identification of recording sites

After completing the recording in one brain area, the recording chamber was removed and new chambers were implanted into other brain areas or into the other hemisphere under similar aseptic surgical procedures. At the end of all experiments, several recording sites were re-approached and marked with electrolytic lesions (100 µA, 10 s). The animals were deeply anesthetized with sodium pentobarbital and perfused with 10% formalin before their brains were removed. The cerebral cortex was cut in coronal sections and stained with neutral red. A location map of the recording sites was constructed on the brain slices by calibrating the lesion site coordinates.

Based on the recording site map and electrophysiological features, we classified the units into A1, PAF, AMY, PU, and GP. The PAF units were differentiated from the A1 units based on anatomic landmark (posterior to the post-ectosylvian sulcus; Fig. 1), tonotopic gradient (BF increases along rostral-caudal direction), and response latency (relatively longer). The AMY units were located in a deeper area and had a lower spontaneous firing rate compared with that of the PU and GP units. The GP units were medial to the PU units and showed the highest spontaneous firing rates.
